# Irregular and suppressed elastic deformation by a structural twist in cellulose nanofibre models

**DOI:** 10.1038/s41598-020-80890-1

**Published:** 2021-01-12

**Authors:** Kojiro Uetani, Takuya Uto, Nozomu Suzuki

**Affiliations:** 1 The Institute of Scientific and Industrial Research, Osaka University, Mihogaoka 8-1, Ibaraki-shi, Osaka, 567-0047 Japan; 2 Organization for Promotion of Tenure Track, University of Miyazaki, Nishi 1-1 Gakuen Kibanadai, Miyazaki, 889-2192 Japan; 3 Department of Molecular and Macromolecular Chemistry, Graduate School of Engineering, Nagoya University, Nagoya, 464-8603 Japan

**Keywords:** Structural properties, Computational science

## Abstract

The elastic responsiveness of single cellulose nanofibres is important for advanced analysis of biological tissues and their use in sophisticated functional materials. However, the mechanical responsiveness derived from the twisted structure of cellulose nanofibres (CNFs) has remained unexplored. In this study, finite element simulations were applied to characterize the deformation response derived from the torsional structure by performing tensile and bending tests of an unconventionally very long and twisted rod model, having the known dimensional parameters and properties of CNFs. The antagonistic action of two types of structural elements (a contour twist and a curvilinear coordinate) was found to result in an irregular deformation response but with only small fluctuations. The contour twist generated rotational displacements under tensile load, but the curvilinear coordinate suppressed rotational displacement. Under bending stress, the contour twist minimized irregular bending deformation because of the orthotropic properties and made the bending stress transferability a highly linear response.

## Introduction

Cellulose nanofibres (CNFs), which are the building blocks of the body tissues of plants and animals, have been attracting attention as the next generation of environmentally friendly nanomaterials owing to their many unique structures and properties. In particular, the mechanical properties are considered to be some of the most important properties of CNFs. Because of the extended-chain crystals of cellulose I type^1^, CNFs possess excellent mechanical properties, including an elastic modulus of 140 to 150 GPa^2^, strength of 2 to 6 GPa^3^, and a coefficient of linear thermal expansion of 0.6 ppm/K^4^, which have promoted their use in various high performance polymer nanocomposites^5–7^ and fibrous assemblies^8–10^. In these materials, the CNFs have been assumed to be ideal elastic rods based on their fibrous shapes, and composite theories for a cylindrical elastic rod have often been applied^5,11^. However, a single CNF has recently been demonstrated to have a right-handed twist structure through high-resolution microscopic analyses^12–14^. It is not clear whether the twisted fibres exhibit the ideal and regular elastic behaviour. An accurate understanding of the relationship between the basic structures and elastic deformation responses of CNFs is of utmost importance to analyse the complicated biostructures, as well as to further improve the mechanical performance in various materials. In particular, it is a great challenge to clarify the elastic deformation response at long scales beyond the torsional period.

Wooden CNFs with a thickness of 2–3 nm obtained by nanofibrillation of wood cell walls have a “right-handed twist”^12^. The twisted CNFs have a torsional period of ~ 232 nm^13^, and the period tends to shorten as the surface charge density increases^14^. A feature of the twisted CNF is that the crystal *c* axis is itself twisted^15^, which is fundamentally different from macroscopic architectures, such as stone pillars and buildings, in which only the contour shape is arranged in a twisted shape, or the coil springs, in which the twisting axis is not aligned with the centre of coil component. However, it is extremely difficult to perform mechanical tests on a single CNF that is much longer than the torsional period. In electron microscopy, CNFs with much longer length than the torsional period do not fit into one field of view, and the CNFs, which are organic substances, are easily damaged by electron beams. Therefore, three-point bending tests in short spans of ~ 250 nm using atomic force microscopy^2^ and probabilistic analysis with ultrasonic fragmentation (sonicated to ~ 300 nm for wood CNFs)^3^ have previously been performed to investigate the mechanical properties of single CNFs. However, mechanistic tests in an extremely long span to account for the torsional effects of the CNF are practically difficult and have not been performed.

The twisted structure of CNFs has been understood as a property of cellulose crystal structure polymorphism. Previously, the twisted structure of cellulose nanocrystals has been mainly predicted through molecular dynamics (MD) simulations^16–18^. The origin of the twist is attributed to the intramolecular and intermolecular hydrogen bonds^19^, van der Waals interactions^20^, cross-sectional area or diameter of the crystals^16,21,22^, and spontaneous twisting of the molecular chain sheets^23,24^. A MD/density functional theory (DFT) study revealed that crystal models of cellulose I exhibit a larger amount of twisting than other polymorphs, suggesting that cellulose II crystals are difficult to twist^23^. The torsion-derived mechanical properties have rarely been considered because previous all-atom MD simulations could only treat very small crystal models with an upper length limit of ~ 50 nm owing to balancing issues of the computational load and accuracy of the intermolecular interactions^23–26^. In addition, there is an inherent disadvantage in all-atom MD-based mechanics calculations that the obtained data are not unique because of the vague definition of the atomic cross-sectional area^27^. Therefore, it is still a great challenge to separately consider the twisting mechanism and the mechanical characteristics derived from the twisted structure based on cellulose crystals. To verify the linearity of the elastic deformation response, we believe that universal understanding of the mechanical characteristics of sufficiently long torsional rod models that are not constrained by the molecular structure of cellulose is vital. To technically handle much longer CNF models than one-round twist (232 nm)^13^, we consider that finite element simulations without atomic information is the only practical option at the present stage.

In the present study, we aim to characterize the elastic deformation response owing to the twisted rod structure of modelled CNF. We performed tensile and bending tests on twisted CNF models at long scales of 1- to 25-round twists (232–5800 nm) by finite element simulation. Based on the reported knowledge about the hexagonal cross-sectional shape and torsion period of wood-derived CNFs, we modelled the twisted rod with a twisted contour and curvilinear coordinate twisting of the coordinate of the cross-sectional direction, where the orthotropic physical properties, including the stiffness tensors, shear modulus, and Poisson’s ratio, are linked. To investigate the effect of the torsional structure in detail, we isolated the presence or absence of contour torsion and curvilinear coordinates. In addition, the evolution of the area moment of inertia for the twisted CNF model was calculated to characterise the bending properties. We then revealed that the structurally twisted CNF model had irregularity of elastic deformation with inherent random yet small fluctuations.

## Results

### Tensile response of the twisted CNF model

**Figure 1:**
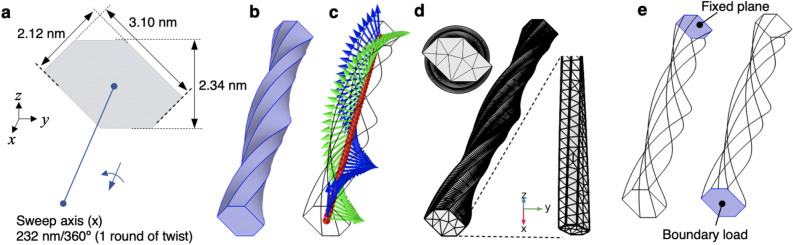
Mathematical modelling of a twisted CNF. (**a**) A hexagonal cross-section assuming an 18-strand crystal model was swept clockwise around the centre of gravity in the normal direction (*x* axis) to create the right-handed twist contour (**b**). (**c**) Torsion of the crystal axis was reproduced by clockwise curvilinear coordinate of the second basis vectors in the *y* and *z* axes, where the orthotropic physical properties of cellulose I*β* crystals are linked. (**d**) A fine tetrahedral mesh was set up in the model. (**e**) The back end was fixed, and a tensile load in the *x*-axis direction or a bending load in the − *z* direction was applied to the front end. The simulation model figures were exported by COMSOL Multiphysics 5.4 (https://www.comsol.jp).

To reveal the twist-derived deformation feature of long single CNF models, finite-element analysis with typical coarse-grained simulations was performed using COMSOL Multiphysics version 5.4 (COMSOL Inc., Stockholm, Sweden). A twisted CNF model with a hexagonal cross-section was constructed with reference to an 18-strand crystal model (Figs. 1a,b, and S1)^28^. The structural torsion of the crystal axis of the CNF was reproduced by setting the curvilinear coordinates in the *y* and *z* directions twisted around the *x* axis, where the orthogonal properties, such as the stiffness tensor, shear modulus, and Poisson’s ratio of cellulose I*β* crystals (described in the Methods section), are linked (Fig. 1c).

By setting the tetrahedral quadratic element meshes with the mesh sizes of 0.464–46.4 nm and the narrow region resolution of 1 to generate a fine-mesh, we adopted 82,685 meshes per 10-round twist in terms of both computational load and convergence. (Fig. 1d). Prior to the main test, we confirmed beforehand that the mesh size dependence of the mechanical displacement did not occur in this setup because the tensile displacement in each orientation remained almost constant when the total tetrahedral mesh number of the 10-round twist model was changed from about 10,000 to about 400,000 in steps. A boundary tensile load in the *x*-axis direction was applied along the *x* axis at the front end surface to perform the mechanical simulation (Fig. 1e). The load was set to 1 nN, which corresponded to stress of 176.7 MPa as calculated using the cross-sectional area of 5.659 nm^2^ for the CNF model.

**Figure 2:**
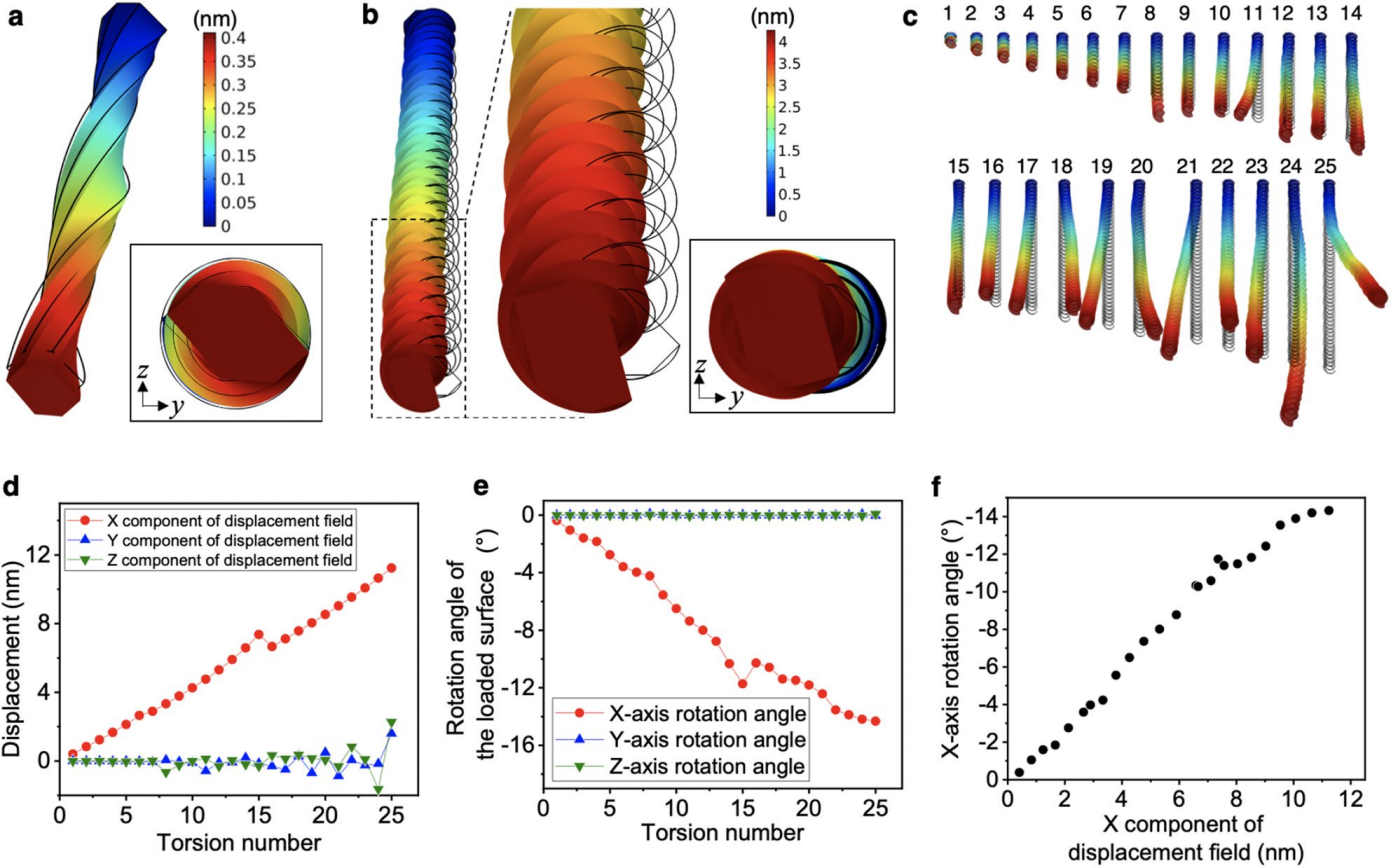
Mechanical simulation of a twisted CNF model. Displacement behaviour of the (**a**) 1- and (**b**) 10-round twist models, with 1 nN load from the original position indicated by the black lines. The displacement in the images for (**a**) and (**b**) was enlarged by 20 and 10 times for easy viewing, respectively. The inserts in both (**a**) and (**b**) are a cross-sectional view of the load plane. The colour bars indicate the total displacement. (**c**) Independent results of mechanical simulations for 1- to 25-round twist models with 1 nN load. The displacement in the image was enlarged by 10 times for easy viewing. (**d**) Displacements and (**e**) rotation angles in the *x*, *y*, and *z* directions plotted against the torsion number. (**f**) *x*-axis rotation angle plotted against the displacement in the *x* direction, showing a roughly inversely proportional relationship. The simulation model figures were exported by COMSOL Multiphysics 5.4 (https://www.comsol.jp).

When a boundary tensile load was applied to the 1-round twist model, the entire model extended in the direction of the load, and the hexagonal cross-section was slightly rotated within the *y*–*z* plane (Fig. 2). Similar results were observed for the 10-twist model with a length of 2320 nm (Fig. 2b). The right-handed model induced left-handed rotational displacement. In addition, the complete image of the model revealed that the model was clearly curved from the original position. The fact that stretching along the *x* axis resulted in rotational deformation in the *y*–*z* plane clearly indicates the inherent irregularity of the deformation exhibiting an irregular load transfer manner.

To confirm that the mechanical irregularity depends on the structural twist, the relationship between the twist and the deformation behaviour was further investigated. A large stress distribution was observed for the twisted CNF model, while a hexagonal rod without twisting showed uniform stress of 176.7 MPa at all sites and did not rotate or bend (Figure S2). In addition, when left-handed twist models were used, the direction of the bending and the rotational displacement were reversed relative to the right-handed model (Figure S3). We also found that the twisting model with a star or triangle cross-sectional shape showed similar bending and rotational displacement to the hexagonal CNF model (Figure S4). Based on these results, we concluded that the twisted shape itself is the basis for expression of irregular rotational deformation.

To highlight the torsional cycle dependence of the deformation behaviour, dynamics simulations were performed for individual models with 1–25 torsion cycles. The deformation amount increased as the torsion period increased, and slight bending and large rotational displacement were observed for all of the models (Fig. 2c). However, the direction of the bending displacement was random and appeared to show no trend. Conversely, the rotational displacements were left-handed to the *x* axis for every model because they were all right-handed models (Figure S3).

To quantitatively evaluate these behaviours, the coordinates of the centre of gravity in the loaded plane after the deformation were extracted and the displacement was separately plotted for the *x*, *y*, and *z* components (Fig. 2d). The elongation in the *x* direction increased with increasing number of twisting cycles; however, the deformation in the *y* and *z* directions was completely random and showed no trend. Regarding the *x*-displacement jump in the 15-round twist model, we repeated the tests in fine steps using the model with non-integer twist numbers and found that the total number of meshes increased rapidly for the 15.55-twist model. The number of mesh partitions per twist length was found increased from about 7408 and 7511 for the 14-round and 16-round twist model, respectively, to ~ 7989 for the 15-round twist model. Although the setting of meshing configuration parameters was not changed, we suspect that the way the mesh was divided may automatically have changed slightly after the 15-round twist model. Therefore, we believe that the displacement jump at 15-round twist model is the result of differences in the way the mesh is divided rather than physical properties. However, the conclusion that the irregular rotational displacements occurred in all models remains the same, and we clearly concluded that a twisted rod do not behave in the same regular way as an untwisted rod. In addition, no mesh size dependence was observed in *y*- and *z*-displacement (Fig. 2d), suggesting that the bending displacements expressed in terms of *y*- and *z*-displacement stay within the range of the calculated fluctuations. Conversely, the rotational displacement significantly increased with the twisting period only around the *x* axis, and almost no rotation around the *y*- and *z*-axes was observed (Fig. 2e). Although the displacements along the *x* axis and the rotational displacements around the *x* axis were roughly inversely proportional, they were not perfectly linear and showed random fluctuating irregularity (Fig. 2f).

The reproducibility of the mechanical simulation may be a problem as the twist period increases. Therefore, the dynamics calculation for the 25-twist model was separately performed 100 times, and the barycentric coordinates of the load surface after deformation were extracted and plotted on a three-axis graph (Figure S5). The *x* component of the displacement showed no variation, and the *y* and *z* components showed slight variation. The total displacement was 11.579 ± 0.002 nm, and the *x*, *y,* and *z* components of the displacement were 11.238 ± 0.000 nm, 1.586 ± 0.008 nm, and 2.291 ± 0.008 nm, respectively. Because the variations were extremely small with respect to the total displacement, the reproducibility was considered to be high. The anisotropy of the *y*- and *z*-displacements in this long model was thought reflecting the asymmetric cross-sectional geometry and the anisotropy of the set stiffness tensors, Poisson's ratio, and shear modulus.

**Figure 3:**
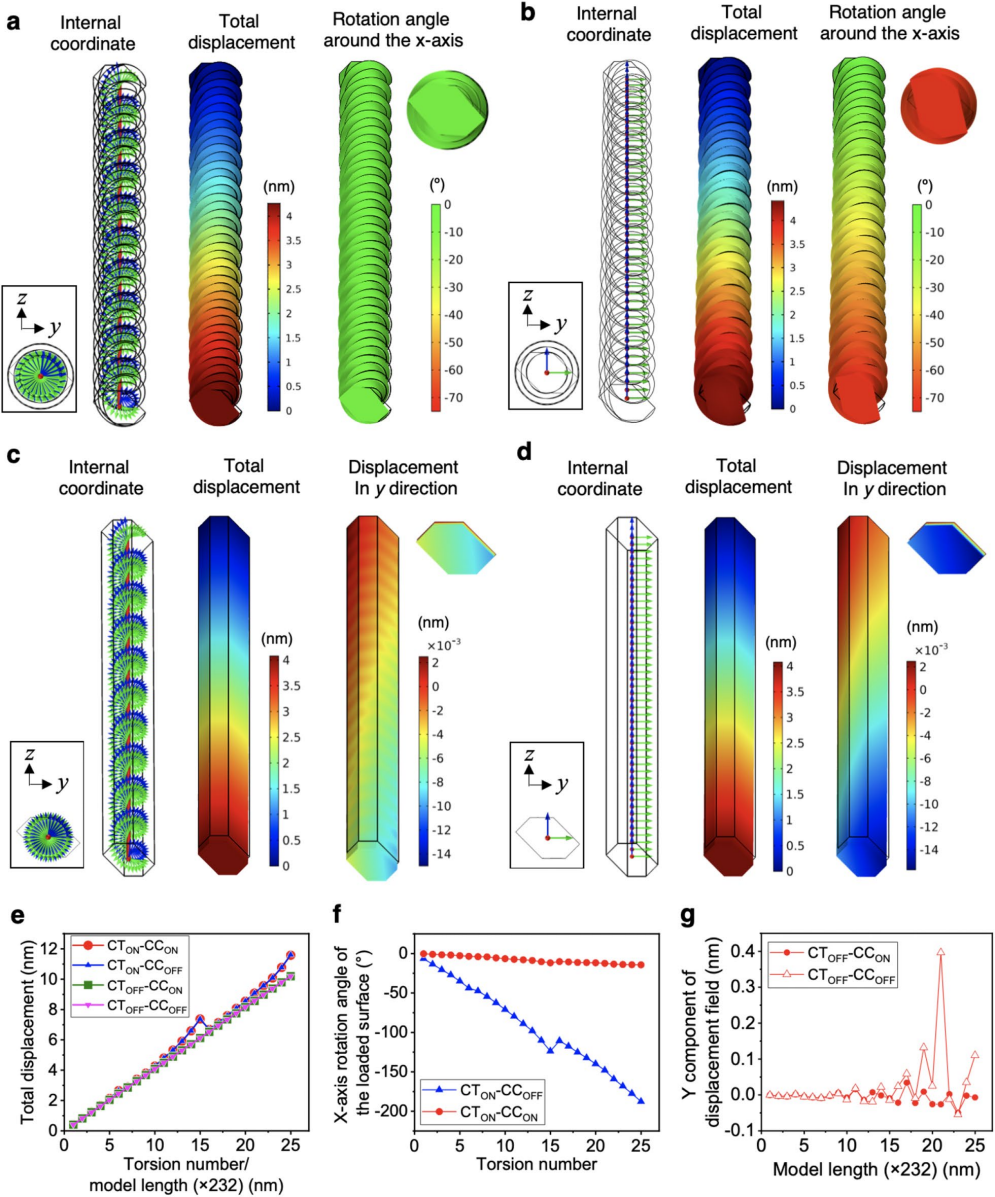
Effects of the contour twist and curvilinear coordinates on tensile deformation. Internal coordinates, total tensile displacement distributions, and the corresponding rotational displacement distributions around the *x* axis for the typical 10-round twist or equivalent 2320 nm length model with (**a**) contour twist ON and curvilinear coordinate ON (CT_ON_-CC_ON_), (**b**) CT_ON_-CC_OFF_, (**c**) CT_OFF_-CC_ON_, and (**d**) CT_OFF_-CC_OFF_. (**e**) Total displacements for the 1- to 25-round twist models or models of equivalent lengths with four different combinations of contour twisting and curvilinear coordinate. (**f**) Difference in rotational deformation around the *x* axis between the CT_ON_-CC_ON_ and CT_ON_-CC_OFF_ models. (**g**) Difference in deformation along the *y* axis between the CT_OFF_-CC_ON_, and CT_OFF_-CC_OFF_ models. The simulation model figures were exported by COMSOL Multiphysics 5.4 (https://www.comsol.jp).

To clarify the structural factors of the irregular tensile deformation responses for the twisted CNF models shown in Fig. 2, we separated the contour twist and curvilinear coordinate which is the twist of the internal coordinate. We produced four different combinations with the presence (ON) and absence (OFF) of a contour twist (CT) and curvilinear coordinate (CC): contour twist ON and curvilinear coordinate ON (called CT_ON_-CC_ON_), CT_ON_-CC_OFF_, CT_OFF_-CC_ON_, and CT_OFF_-CC_OFF_, and performed tensile tests applying 1 nN load, and the results are shown in Fig. 3. The CT_ON_-CC_ON_ model (Figs. 2 and 3a) corresponds to an actual CNF with structural torsion, while the CT_OFF_-CC_OFF_ model (Fig. 3d) corresponds to a general uniform orthotropic material with no torsion.

Interestingly, by comparing Fig. 3a and 3b, for the models with a contour twist, the presence of the curvilinear coordinate suppressed rotational deformation around the *x* axis. On the other hand, in the case of the straight models without a contour twist, the bending deformation was suppressed by the curvilinear coordinate, especially in the *y* direction, although small deformation fluctuation corresponding to the curvilinear coordinate was observed (Fig. 3c,d). The bending deformation shown by the straight model without the curvilinear coordinate was thought to be derived from the orthotropic physical properties having large anisotropy based on cellulose I*β* crystals^29^.

When the model lengths were changed from 232 to 5800 nm (corresponding to 1- to 25-round twist) step by step, the total tensile displacement changes for the contour-twisted models were not linear like the straight models (Fig. 3e), although this result includes the influence of the mesh segmentation method described above. Within the contour-twisted models, the existence of the curvilinear coordinate significantly decreased the rotational deformation (Fig. 3f). Deformation suppression by the curvilinear coordinate was also observed for the straight models without a contour twist. Although the bending deformation extracted by the *y*-direction displacements tended to be smaller for the models with the curvilinear coordinate than those without the curvilinear coordinate, while they showed fluctuations (Figs. 3g and S6).

### Bending response for the twisted CNF model

**Figure 4:**
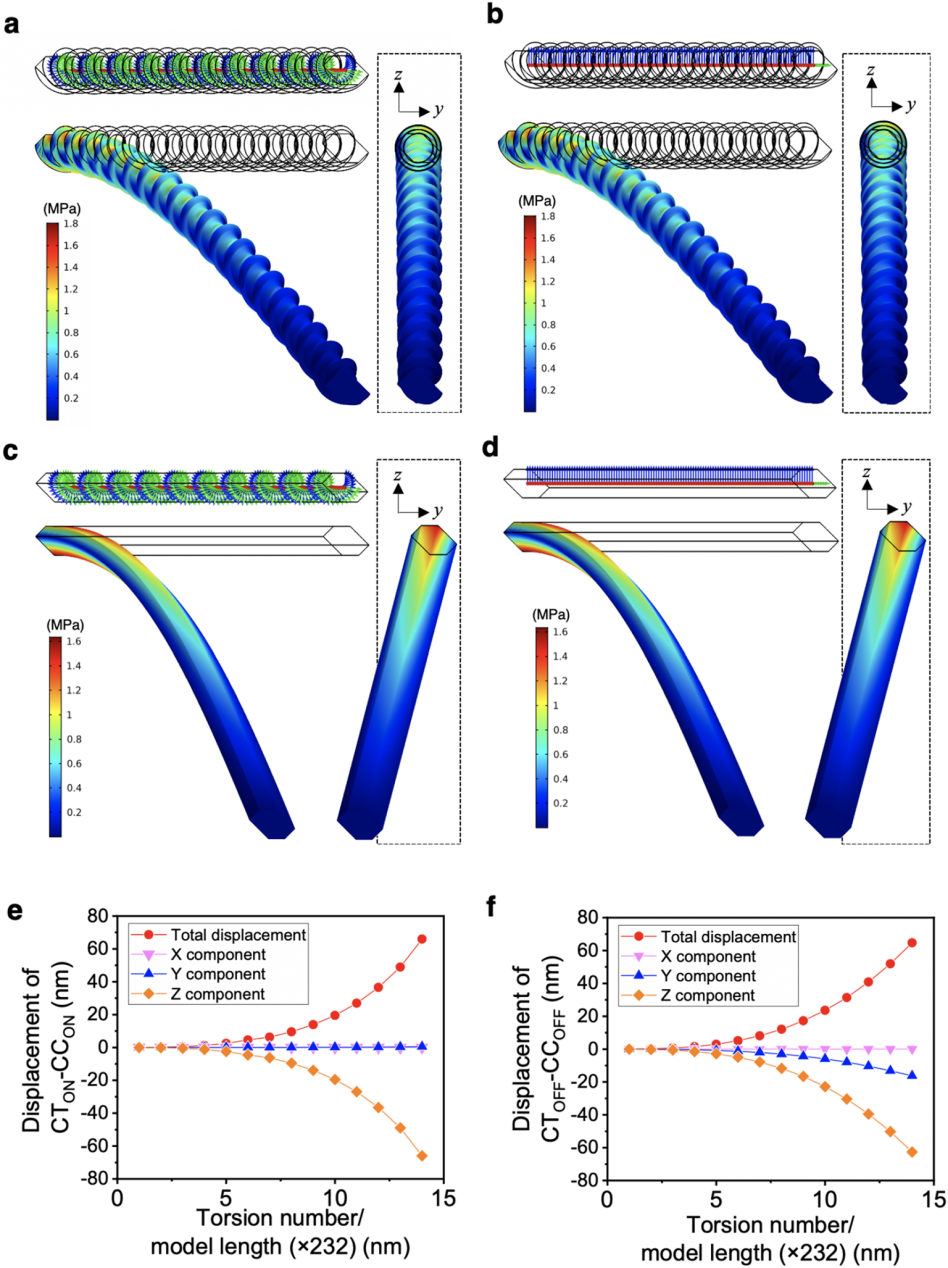
Effects of a contour twist and curvilinear coordinates on bending deformation. Internal coordinates and von Mises stress distributions for typical 10-round twist or equivalent 2320 nm length models with (**a**) CT_ON_-CC_ON_, (**b**) CT_ON_-CC_OFF_, (**c**) CT_OFF_-CC_ON_, and (**d**) CT_OFF_-CC_OFF_. Displacements in each direction for the 1- to 14-round twist models (corresponding to 232 to 3248 nm in length) of (**e**) CT_ON_-CC_ON_ and (**f**) CT_OFF_-CC_OFF_. The simulation model figures were exported by COMSOL Multiphysics 5.4 (https://www.comsol.jp).

In addition to the tensile behaviour, the bending deformation behaviour is also important for flexible CNFs. We performed bending tests by applying 1 fN load in the − *z* direction at the load surfaces for the four different models CT_ON_-CC_ON_, CT_ON_-CC_OFF_, CT_OFF_-CC_ON_, and CT_OFF_-CC_OFF_ (Fig. 4a–d). Most strikingly, the model without a contour twist showed large displacements in the *y* direction in addition to the loading direction (− *z* direction). This displacement, which differed from the loading direction, was independent of the presence or absence of curvilinear coordinates. The models with a contour twist appeared to show displacement only in the loading direction, and they were more likely to show linear deformation behaviour.

When the model lengths were changed from 232 to 3248 nm (corresponding to 1- to 14-round twists) step by step, the CT_ON_-CC_ON_ model showed displacements in the loading direction (− *z* direction) that were nearly identical to the total displacement and showed little displacement in the *x* or *y* direction (Fig. 4e). In other words, we concluded that the bending stress transferability of the CT_ON_–CC_ON_ model responded with high linearity. However, the CT_OFF_-CC_OFF_ model clearly showed irregular displacements in the *y* direction in addition to the loading direction (Fig. 4f). This irregular response was also thought to be derived from the orthotropic properties having large azimuthal anisotropy based on cellulose I*β* crystals^29^, in a similar manner to the tensile test. Thus, the contour twist was demonstrated to suppress irregular bending deformation owing to anisotropy of the properties. The presence or absence of curvilinear coordinates did not seem to have much effect on bending deformation, unlike tensile deformation. Exactly the same behaviour was observed in the bending test with the *y*-direction load, although the amount of deformation was different from the − z-direction  load test shown in Fig. 4. In addition, the 14-round twist model is the upper limit length that can be calculated in our system, and the calculations did not converge at lengths longer than that because of the large displacements. The actual CNF can be approximated by the CT_ON_-CC_ON_ model, and depending on the crystal thickness, surface conditions or drying conditions, it approaches the torsion-free CT_OFF_-CC_OFF_ model^14,15^. The twisted CNF models were found to be closer to the linear response material owing to averaging of the nonlinear curvature caused by the anisotropy of the properties.

**Figure 5:**
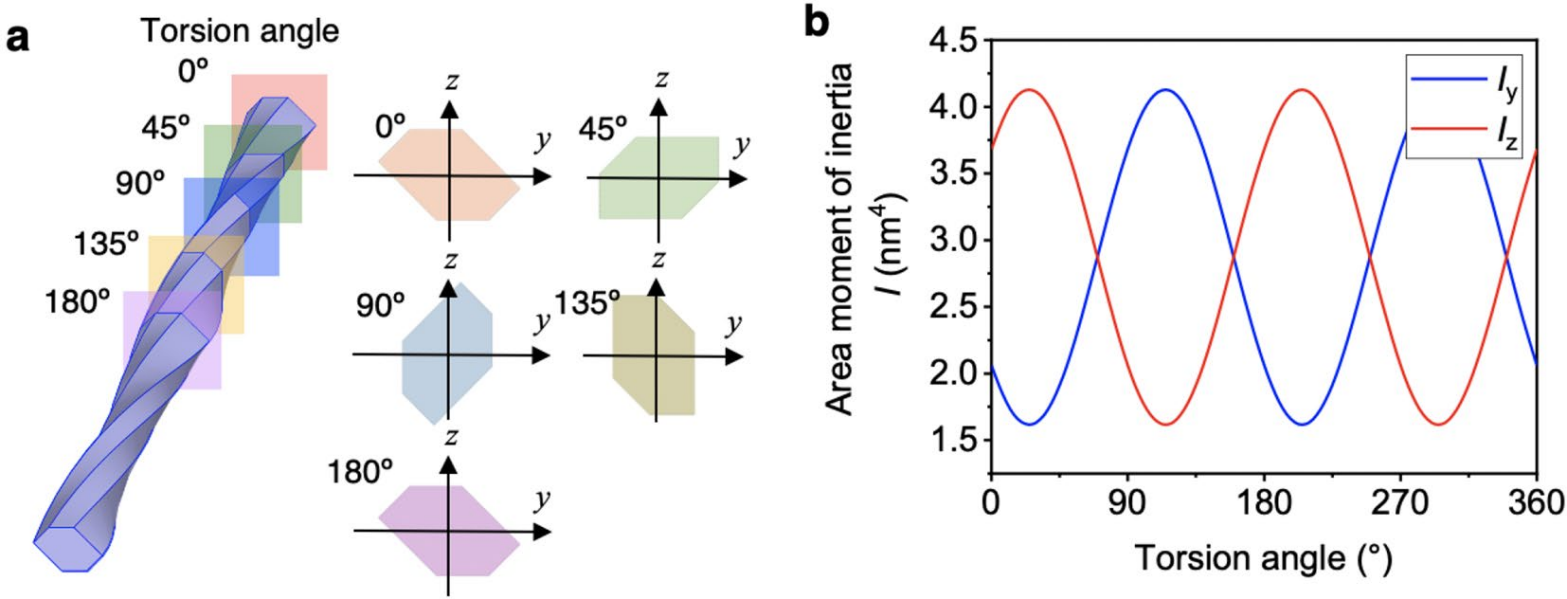
Area moment of inertia periodically modulated by a contour twist. (**a**) Sectional change in the relative torsion angle. (**b**) Area moment of inertia relative to the *y* and *z* axes (*I*_y_ and *I*_z_, respectively), showing a periodic change with respect to the torsion angle. The twisted model figures were exported by COMSOL Multiphysics 5.4 (https://www.comsol.jp).

To clarify why the contour twist averages the anisotropy of the properties, we focused on the changes in the cross-sectional structure (Fig. 5a). The contour-twisted model showed that the cross-sectional directions varied along the CNF for a constant bending loading direction. The bendability of the rod is described by the area moment of inertia. When the area moment of inertia for the twisted CNF model was calculated for the *y* and *z* direction, $I_{y} = \mathop \smallint \limits_{s} z^{2} {\text{d}}s$ and $I_{z} = \mathop \smallint \limits_{s} y^{2} {\text{d}}s$, respectively, *I*_y_ and *I*_z_ showed remarkable periodicity (Fig. 5b, see also Appendix S1 in the Supporting Information for derivation of the general area moment of inertia for the CNF model). Namely, the bendability periodically changed. This periodicity is thought to average the anisotropy of the properties and make the response to deformation more linear. By contrast, a model without torsion has a constant area moment of inertia, so the internal anisotropy of the properties is more likely to be manifested.

## Discussion

In the structurally twisted CNF (CT_ON_-CC_ON_) model, the antagonistic action of two types of structural elements (a contour twist and a curvilinear coordinate) was found to result in an irregular deformation response but with only small fluctuations. The contour twist generates rotational displacements under tensile load, but the curvilinear coordinate was found to suppress the rotational displacements. The curvilinear coordinate also suppresses bending deformation under tensile load owing to the orthotropic properties of the cellulose crystal for the untwisted models, and the deformation irregularity only shows small fluctuations. Conversely, under bending stress, the contour twist minimises deformation response different from the direction of loading owing to the orthotropic properties and makes the bending stress transferability a highly linear response. The homogenisation of the bending response is derived from the averaging of the orthotropic properties by the periodic variation of the area moment of inertia. We believe that we extracted uniform trends that are common to all models with different torsion numbers, although some results for the model with large torsion number included the effect automatic settings such as the mesh segmentation method by the COMSOL software. While it has been reported that coarse-grained MD can simulate models with lengths as long as 1200 nm^22^, our finite element calculations have a significant advantage in dealing with lengths until 25-round twists (corresponding to 5800 nm) in tensile tests and 14 round twists (corresponding to 3248 nm) in bending tests, and we have succeeded in clarifying the irregular characteristic of pure torsional structures that do not take into account the atomic information. The characteristic mechanical responsiveness derived from the torsional structure revealed in this study could be useful for more precise analysis and characterisation of complex biological tissues and highly sophisticated functional materials.

## Methods

To construct a mathematical model of a CNF, the hexagonal cross-section of the CNF was drawn on the *y–z* plane with reference to an 18-strand crystal model (Figs. 1a and S1)^28^. The centroid of the hexagon was set as the origin, and the geometry was swept perpendicular to the plane of the hexagon (i.e., along the *x*-axis direction) from the origin to 232 nm in length (twisting angle of ~ 1.55°nm^−1^)^13^. As shown in Fig. 1a,b, the hexagonal cross-section was rotated clockwise for 360° to form the twisted geometry. Next, the torsion of the crystal axis of the CNF was reproduced by curvilinear coordinate twisting of the second basis vectors **v**_2_ clockwise in the *y* and *z* axes, where the physical properties are linked as follows (Fig. 1c):

$${\mathbf{v}}_{2} = \left( {\begin{array}{*{20}c} x \\ y \\ z \\ \end{array} } \right) = \left( {\begin{array}{*{20}c} 0 \\ {\cos \left( {\frac{{x \left[ {{\text{nm}}} \right]}}{{232 \left[ {{\text{nm}}} \right] }} \times 2{\uppi }} \right)} \\ {\sin \left( {\frac{{x \left[ {{\text{nm}}} \right]}}{{232 \left[ {{\text{nm}}} \right]}} \times 2{\uppi }} \right)} \\ \end{array} } \right)$$

We then approximated the system as orthotropic using the stiffness tensor with *E*_*xx*_ = 100.5 GPa, *E*_*yy*_ = 75.1 GPa, and *E*_*zz*_ = 25.6 GPa, the shear modulus with *G*_*xy*_ = 15.5 GPa, *G*_*yz*_ = 3.4 GPa, and *G*_*xz*_ = 2.9 GPa, and the Poisson’s ratio with *ν*_*xy*_ = 0.81, *ν*_*yz*_ = 0.17, and *ν*_*xz*_ = 0.60 for cellulose I*β* crystals^29^. The density was set to 1600 kg m^−3^ according to Daicho et al.^30^. After finely dividing into tetrahedral meshes (Fig. 1d), the backside end surface was constrained to the original position.

A boundary load direction was applied at the front end surface to perform the mechanical simulation (Fig. 1e). For the tensile test, the load was set to 1 nN on the *x* axis, which corresponded to a stress of 176.7 MPa as calculated using the cross-sectional area of 5.659 nm^2^ for the CNF model. For the bending test, 1 fN was set on the minus *z* axis. The displacements in each direction by the mechanical tests were calculated with a domain point probe set at the centre of gravity of the load plane. The rotational displacements were calculated as the average rotation angle with the loaded surface as the boundary probe.

## Supplementary information

Supplementary Information 1.
