# Supplementary material for: Irregular and suppressed elastic deformation by a structural twist in cellulose nanofibre models
Source: Sci Rep. 2021 Jan 12;11:790. doi: 10.1038/s41598-020-80890-1 (PMC7803750; doi:10.1038/s41598-020-80890-1)
Supplement: Supplementary file 1 — Supplementary Information 1. [file 41598_2020_80890_MOESM1_ESM.docx]

Supplementary Information for

**Irregular and Suppressed Elastic Deformation by a Structural Twist in Cellulose Nanofibre Models**

**Kojiro Uetani**1,***, Takuya Uto**2**, and Nozomu Suzuki**3

^1^The Institute of Scientific and Industrial Research, Osaka University, Mihogaoka 8-1, Ibaraki-shi, Osaka 567-0047, Japan

^2^Organization for Promotion of Tenure Track, University of Miyazaki, Nishi 1-1 Gakuen Kibanadai, Miyazaki 889-2192, Japan

^3^Department of Molecular and Macromolecular Chemistry, Graduate School of Engineering, Nagoya University, Nagoya 464-8603, Japan


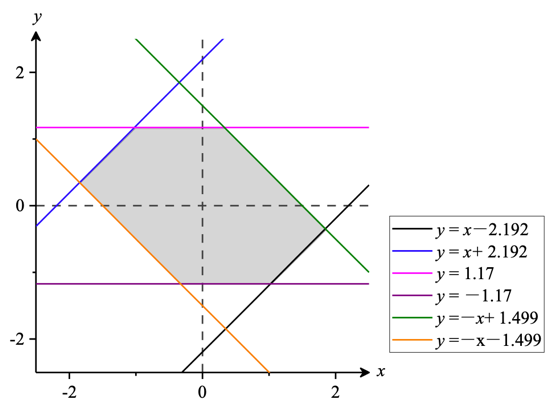


Figure S1. Coordinate representation of hexagonal cross section for a CNF model based on an 18-strand crystal model.^1^ The centroid set at the origin. The scale is in nanometres. All the mathematical models for the twisted CNF created on COMSOL used this cross-sectional shape.


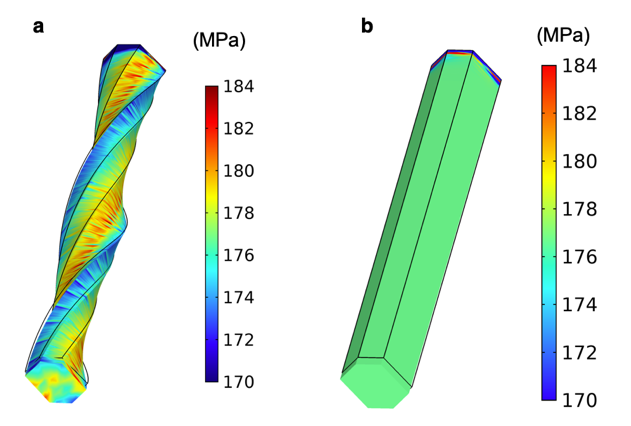


**Figure S2. Von Mises stress distribution** with a 1-nN load exhibited by a one-round twisted CNF model (**a**) and an untwisted hexagonal rod (**b**). The large stress distribution is observed in the twisted model whereas the straight rod shows a uniform stress of 176.7 MPa as calculated using a cross sectional area of 5.659 nm^2^ at all sites. The simulation model figures were exported by COMSOL Multiphysics 5.4 (https://www.comsol.jp).

**
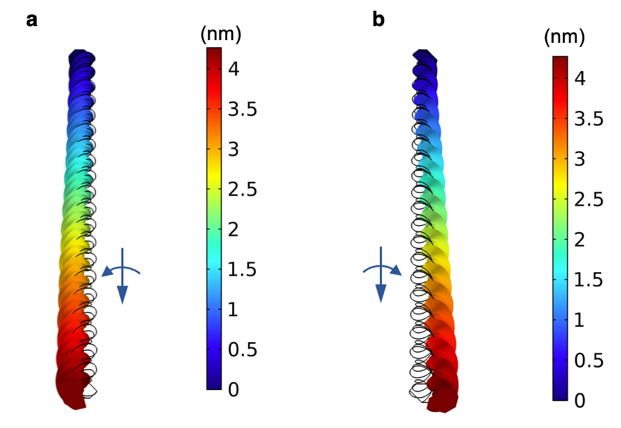
**

**Figure S3. Verification of right and left differences in deformation behaviour using 10-round torsion model.** The right-handed model (**a**) produced a left-handed rotational displacement, while the left-handed model (**b**) produced a right-handed rotational displacement. From the fact that the directions of the bending displacements also contrasted with each other, it was found that both of the bending and rotational displacements are caused by the twisting shape. The simulation model figures were exported by COMSOL Multiphysics 5.4 (https://www.comsol.jp).


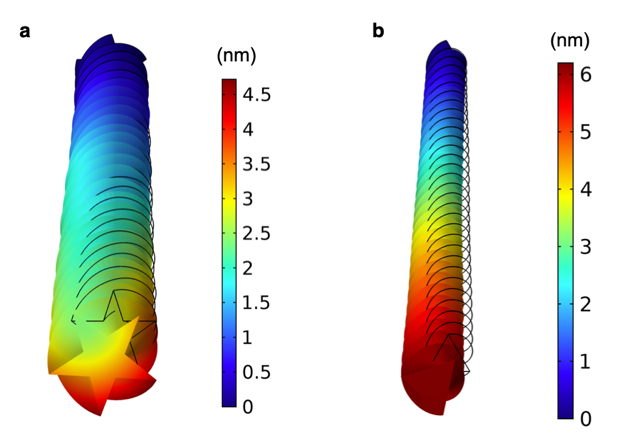


**Figure S4. Displacement behaviour independent of cross-sectional shape.** The right-handed model with (**a**) star-shaped or (**b**) equilateral triangle cross section showed bending and rotational displacement as well as the hexagonal CNF model. The simulation model figures were exported by COMSOL Multiphysics 5.4 (https://www.comsol.jp).

**
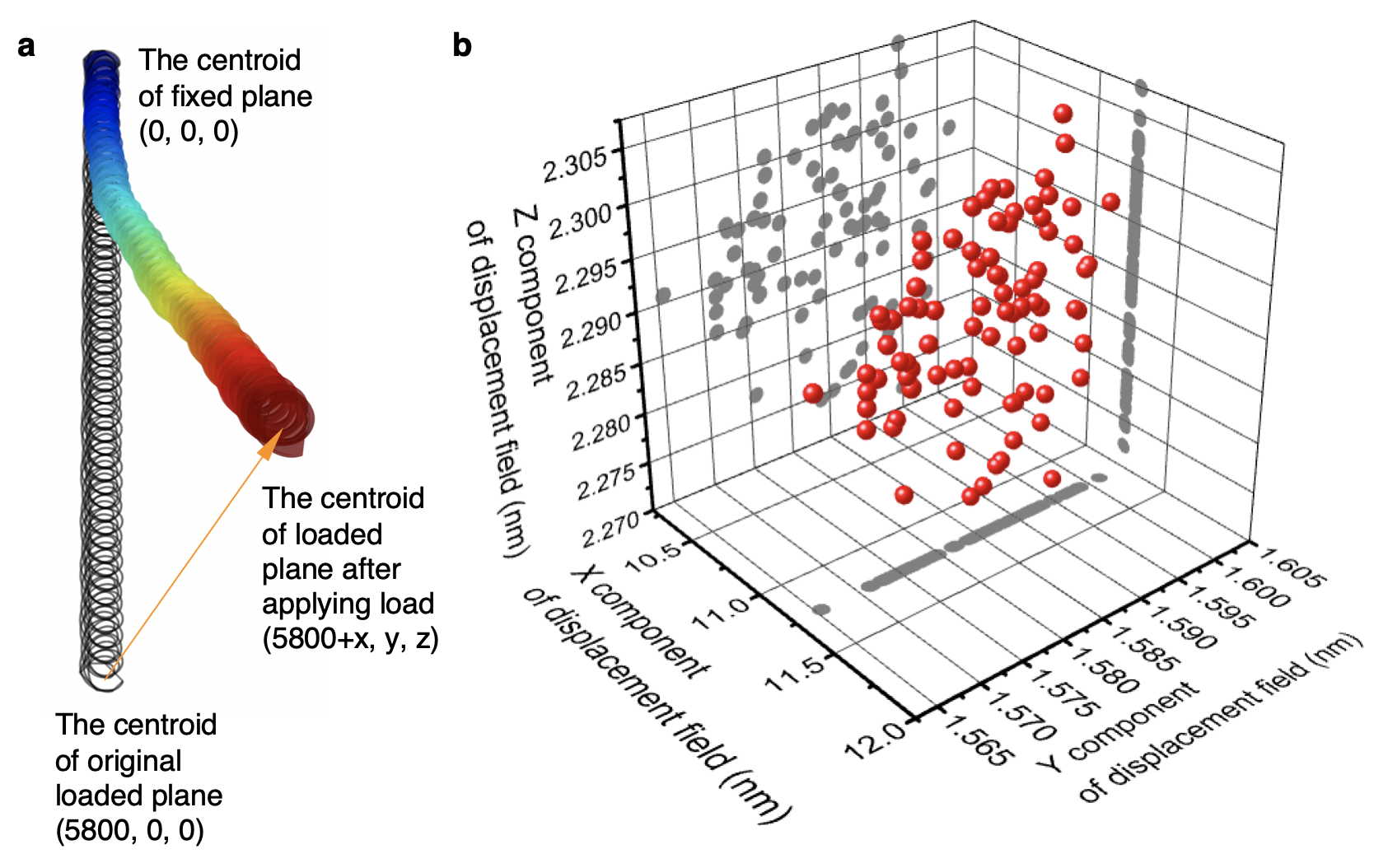
**

**Figure S5. Reproducibility evaluation by 100 COMSOL mechanical simulations with a 25-round twisted CNF model.** (**a**) The centroid coordinates of the loading plane before and after applying a 1 nN load were extracted as displacement fields in the calculation with a CNF model of 25-round torsion. (**b**) Variation of the *x*, *y*, and *z* components of the displacement field in 100 calculations. For a total displacement of 11.579 ± 1.909×10^-3^ nm, the *x*, *y*, and *z* components of the displacement field are 11.238 ± 0 nm, 1.586 ± 7.947×10^-3^ nm, and 2.291 ± 7.912 × 10^-3^ nm, respectively. While the *x* component of the displacement field did not change at all, the *y* and *z* components had slight variations. Because the variation was extremely small relative to the total displacement, the reproducibility of the calculation was evaluated to be high. The simulation model figures were exported by COMSOL Multiphysics 5.4 (https://www.comsol.jp).


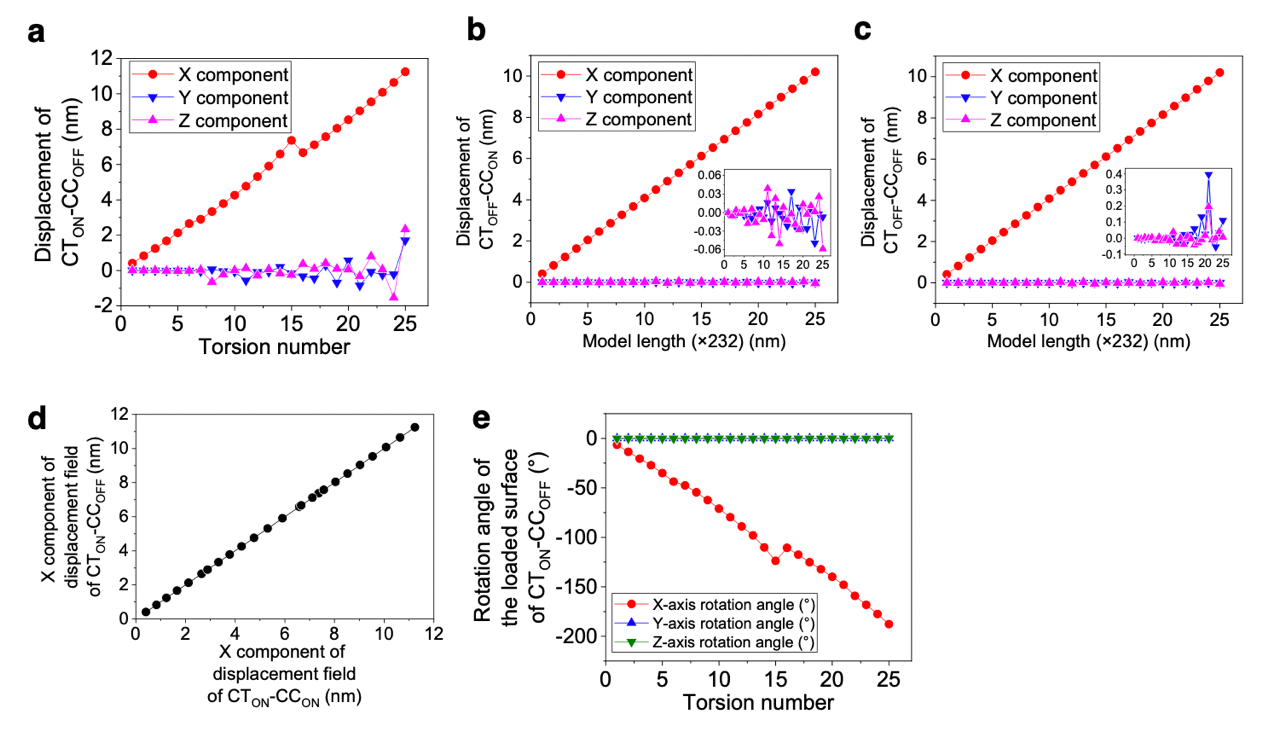


**Figure S6. Tensile deformation results.** The displacements in *x*, *y*, and *z* directions against the torsion numbers from 1 to 25 or equivalent 232- to 5800-nm length model of (**a**) CT_ON_-CC_OFF_, (**b**) CT_OFF_-CC_ON_, and (**c**) CT_OFF_-CC_OFF_. (**d**) Displacement comparison in *x* direction between CT_ON_-CC_ON_ shown in Figure 2d and CT_ON_-CC_OFF_. (**e**) The rotation angles of the CT_ON_-CC_OFF_ model.


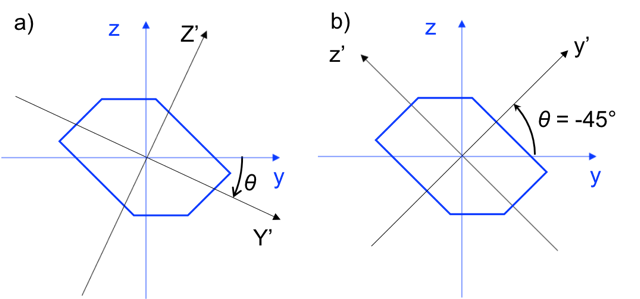


**Fig. S7. Conversion of the coordinates in the cross-section of CNF.**


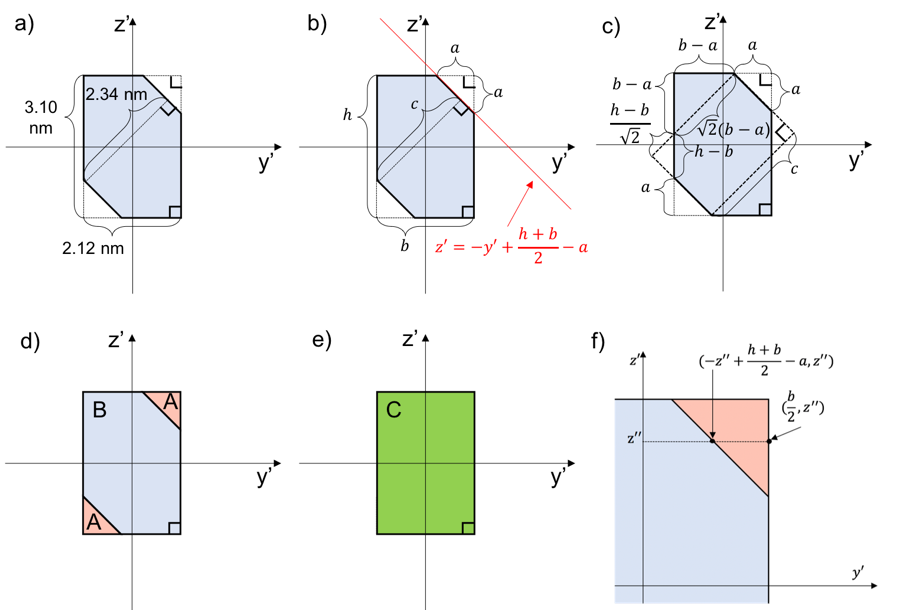


**Fig. S8. The cross sectional geometry of CNF.** (**a**) Length of each edge from the reference^1^. (**b**) Definition of length *a*, *b*, *c*, and *h*. (**c**) Correlation between *c* and a set of three values *a*, *b*, and *h*. From the figure, *c* can be calculated as $\sqrt{2}\left( b-a \right)+(h-b)/\sqrt{2}=(b-2a+h)/\sqrt{2}$. (**d**) Definition of the area A (red part) and B (blue part). (**e**) The definition of the area C (green part) C = A + B. (**f**) The position of two edges of area A at *z*’ = *z*’’.

**Appendix S1.**

**Derivation of the area moment of inertia of the hexagonal cross-sectional shape of twisted CNF model.** Because the helical CNF in Fig. 1 has a right handed structure, the torsion angle in Fig. 5a can be defined as *θ* in Fig. S7a. To make it easy to calculate the area moment of inertia, the coordinates in the cross-section of CNF was converted from *x*- and *y*-axis to *x*’- and *y*’-axis which corresponds to *θ* = −45° in Fig. S7b.

Second moment of areas *I*_y’_ and *I*_z’_ for rectangle C are known to be *bh*^3^/12 and *b*^3^*h*/12, respectively. *I*_y’_ and *I*_z’_ can be calculated by subtracting two separated area A from C as follows.

| $I_{y'}=\int_{B} {z'}^{2}dB=\int_{C} {z'}^{2}dC-2\int_{A} {z^{'}}^{2}dA=\frac{bh^{3}}{12}-2\int_{\frac{h}{2} -a}^{\frac{h}{2}} \int_{-z^{'}+\frac{h+b}{2} -a}^{\frac{b}{2}} {z^{'}}^{2}\mathrm{dy}^{'}dz'$ |  |
| --- | --- |
| $=\frac{bh^{3}}{12}-\frac{1}{12}a^{2}(2a^{2}-4ah+3h^{2})$ | S-1 |

| $I_{z'}=\frac{b^{3}h}{12}-\frac{1}{12}a^{2}(2a^{2}-4ab+3b^{2})$ | S-2 |
| --- | --- |

Because rectangular shape C has axial symmetry along *x*- and *y*-axis, the product of inertia of area is 0. Therefore, the product of inertia of the area for B can be calculated as follows.

| $I_{y'z'}=\int_{B} y'z'dB=\int_{C} y'z'dC-2\int_{A} y^{'}z^{'}\mathrm{dA} =0-2\int_{\frac{h}{2} -a}^{\frac{h}{2}} \int_{-z^{'}+\frac{h+b}{2} -a}^{\frac{b}{2}} y^{'}z^{'}\mathrm{dy}^{'}dz'$ |  |
| --- | --- |
| $=-\frac{1}{12}a^{2}(a^{2}+3bh-2a(b+h))$ | S-3 |

The relationship between Y’ Z’ and y’ z’ coordinates defined in Fig. S7 can be described as follows.

| $\left[ \begin{matrix} Y^{'} \\ Z^{'} \end{matrix} \right]=\left[ \begin{matrix} \cos\left( \theta+\frac{\pi}{4} \right) & -\sin\left( \theta+\frac{\pi}{4} \right) \\ \sin\left( \theta+\frac{\pi}{4} \right) & \cos\left( \theta+\frac{\pi}{4} \right) \end{matrix} \right]\left[ \begin{matrix} y' \\ z' \end{matrix} \right]$=$\left[ \begin{matrix} y'\cos\left( \theta+\frac{\pi}{4} \right)-z'sin(\theta+\frac{\pi}{4}) \\ y'\sin\left( \theta+\frac{\pi}{4} \right)+z'cos(\theta+\frac{\pi}{4}) \end{matrix} \right]$ | S-4 |
| --- | --- |

Thus, the moment of inertia of area at arbitrary angle *θ* can be described as follows.

| $I_{Y'}=\int{Z'}^{2}dA=I_{y^{'}}\cos^{2}\left( \theta+\frac{\pi}{4} \right)+I_{z^{'}}\sin^{2}\left( \theta+\frac{\pi}{4} \right)+I_{{y'z}^{'}}\cos\left( 2\theta\right)$ | S-5 |
| --- | --- |
| $I_{Z'}=\int{Y'}^{2}dA=I_{y^{'}}\sin^{2}\left( \theta+\frac{\pi}{4} \right)+I_{z^{'}}\cos^{2}\left( \theta+\frac{\pi}{4} \right)-I_{{y'z}^{'}}\cos\left( 2\theta\right)$ | S-6 |
| $I_{Y'Z'}=\int X'Y'dA=\frac{1}{2}\left( I_{z^{'}}-I_{y^{'}} \right)\cos\left( 2\theta\right)-I_{{y'z}^{'}}\sin\left( 2\theta\right)$ | S-7 |

**References**

1 Daicho, K., Saito, T., Fujisawa, S. & Isogai, A. The Crystallinity of Nanocellulose: Dispersion-Induced Disordering of the Grain Boundary in Biologically Structured Cellulose. *ACS Appl. Nano Mater.* **1**, 5774-5785 (2018).
